# Research on the effect of different types of short music videos on viewers' psychological emotions

**DOI:** 10.3389/fpubh.2022.992200

**Published:** 2022-10-26

**Authors:** Lin Ma

**Affiliations:** School of Art, Sun Yat-sen University, Guangzhou, China

**Keywords:** short music video, viewer, psychological emotion, audience psychology, emotional impact

## Abstract

There is now widespread agreement that different types of short music videos can cause viewers to have psychological emotions, and significant new findings have been discovered in the study of how music affects listeners' affective reactions. However, there is still controversy regarding research on the inclinations toward behavior and autonomic neurophysiological reactions of musical emotions. The psychological states of viewers of various types of short music videos are yet unknown and require further study. This study investigates how different types of short music videos affect viewers' psychological responses, placing particular emphasis on the following variables: rhythm type (stable rhythm and flow rhythm) and music short video type (narrative, live, and funny). In an experiment, viewers' psychological responses to several short music videos were investigated to determine the impact of different short music video styles and rhythms on musically induced emotions.

## Introduction

Language and music both distinguish human social interaction and are essential components of communication. Music has a profound impact on all aspects of life, including work and study. It also helps individuals connect and communicate emotionally (1). Watching brief music videos allows viewers to both comprehend the makers' intentions and the emotions they hope to evoke as well as experience those emotions firsthand (2, 3). According to Koordeman et al. (4), the process of breaking down musical emotions can be broken down into different levels of individual cognition and experience. The consequences of each level vary in terms of the emotional content and manner of production, and they include musical emotion perception, experience, and musical taste. The relationship between music and emotions has been the topic of much psychological research, according to Fuentes-Sánchez et al. (5), and relevant studies have demonstrated how specific elements of listeners' interactions with music can alter how they perceive and experience musical emotions. To identify the gaps in the theory and measurement techniques of musical emotion perception, the researchers first conducted a thorough literature review from the starting point of musical emotion, mastered the current theoretical research and application, and discovered the theoretical gaps (6). Johnson et al. (7) states that Western classical music cannot explain the emotional responses to Chinese traditional pentatonic modes, and nothing is known about how viewers' emotional responses to various pop music videos' visual components. This study focused on the visual aspects of short music videos to gauge viewers' emotional perception. Research questions and experimental hypotheses on the influence of different features on psychological emotions are proposed to discuss the modes and video types that lack a theoretical foundation. Psychological experiments were designed and conducted to test the experimental hypotheses, address research issues, draw conclusions, and improve theoretical frameworks.

The main contributions of this paper are as follows:

(1) This study investigates how different types of short music videos affect viewers' psychological responses, placing particular emphasis on the following variables: rhythm type (stable rhythm and flow rhythm) and music short video type (narrative, live, and funny).

(2) Compared to narrative and live music short videos, funny music short videos significantly increased the positive emotion in viewers. In comparison with that, the neutral emotion valence induced by the narrative and live music short video was significantly higher. Performance music short videos produced more polarized emotional valence than narrative music short videos. The emotional valence, arousal, vitality, and restlessness in a brief music video were significantly influenced by the rhythm type.

## Mechanism of short music video affecting viewer's psychological emotion

### Short music videos affect viewers' psychological mood

There has been relatively little research on how short videos affect people's perception over the past few decades, despite the fact that music has been shown to influence people's perception and memory of short videos (8). Barney et al. (9) measured the emotional experience of musicians and non-musicians through short videos of musical performances and discovered that both musicians and non-musicians who only listened to music and those who watched music and short videos simultaneously reported having a stronger emotional experience than those who only watched short videos of musical performances. In addition, musicians think that symphonies and quick videos of performances enhance the emotional impact of the music (10). Different kinds of short music videos can affect viewers' emotions and trigger different cognitive or emotional responses. According to Tian and Zhao (11), listening to music while watching movie clips led to lower performance on cognitive tasks and higher mood scores. On cognitive tests, the group that included live performance and music did better. If the short music videos' content is in line with psychological feelings, the viewer will also feel different emotions and has a different experience. In their study, Egidi and Caramazza (12) discovered that the “emotional consistency” rule governs how short music videos' emotional effects affect music-induced feelings. In Egidi's study, music-based short videos were used to elicit either positive, neutral, or negative emotions in the participants. Initially, neutral mood music was combined with positive emotions in the videos after the participants reported feeling positive, while initially neutral mood music was combined with negative emotions in the videos to elicit negative emotions in the participants.

### Music short video type

Since users process both the content of songs and short music videos at the same time, the associative consistency model predicts that their emotional response will be influenced by the relationship between the content of songs and lyrics and the content of short music videos. In addition, the types of music short videos, such as narrative and live performance, will influence different musical perceptions, which will then result in various emotional experiences or cognitive styles (13). As a result, pop short music videos are divided into three categories based on two criteria: whether the narrative in the short music video is related to the lyrics, and whether it is not.

Short music videos with narration: These short music videos tell one or more stories that are essentially complete;

Live short music videos: In this short music video, the photographer performs live;

Funny short music videos: Snack-sized music videos with wacky narratives, animations, or live performances.

## Hypothesis of the influence of short videos of different types of music on viewers' psychological emotions

### Hypothesis of the induced influence of narrative short music videos

Gardstrom et al. (14) asserts that after being stimulated by brief music videos, users' emotional experiences will become more intense and polarized. In addition, the stimulation of brief music videos with a plot can strengthen emotional reaction while impairing cognitive task performance, whereas the stimulation of brief music videos with abstract content can make people require more cognitive processing and, as a result, weaken emotional reaction. As a result, this study puts forth hypothesis 1–3, which states that narrative short music videos are more likely to elicit strong emotional (valence and arousal) responses than performance short music videos.

Hypothesis 1: The emotional valence induced by narrative short music videos is more polarized than performance short music videos.

Hypothesis 2: The emotional arousal level induced by narrative short music videos is more polarized than performance short music videos.

Hypothesis 3: The type of short music videos has an effect on the specific types of emotions induced by short music videos.

### Hypothesis of the induced influence of live short music videos

According to theoretical research on music and performing music rhythm type short video stimulus, including rhythm in music performance type short video stimulation can improve the listener's emotional experience. Ma et al. (15) believed that the kata music short video stable rhythm type has a greater partial positive titer and higher arousal level than the flowing rhythm type on the induced mood. The hypothesis of this study was that the stimulation of live short music videos would not change the differences and trends of rhythmical emotions.

Hypothesis 4: Rhythm type has an effect on the emotion induced by live short music videos. Steady rhythm has more positive valence and higher arousal levels than flow rhythm.

Hypothesis 5: Rhythmic type has an effect on specific emotions induced by live short music videos. Stable rhythm and flow rhythm induce different types of specific musical emotions.

### Hypothesis of the induced influence of funny short music videos

Funny short music videos are a common type of network video, and it is important to understand what makes them so popular. Merry and Silverman (16) investigated the types of emotional reactions that humorous short music videos will elicit from the viewpoint of video-induced emotion and contrasted this short music video form with more conventional short music video types (narrative type and live performance type) induced emotion. Therefore, this study's main hypothesis is that the rhythmic and short-form music features interact to affect emotional experience.

Hypothesis 6: Funny short music videos and rhythm have an interaction on psychological emotions.

Hypothesis 7: Funny short music videos interact with rhythmic music for specific emotions.

## Empirical process

### Research object

Social media and BBS were used to find 25 university students who would take part in the experiment. This study chose the applicants through an online questionnaire to take into account how the subjects' musical training and familiarity with music affected their emotions. The applicants had to list at least one line from the song or scene from the brief music video in the screening questionnaire. Applications with an overall accuracy rate of <60% will not be taken into consideration. Furthermore, all subjects were right-handed, healthy, and free from either short- or long-term hearing loss. The basic information of the research object is shown in Table 1.

**Table 1:** Basic information on the research object.

**Variable**	**Options**	**Number of samples**	**Proportion (%)**
Age	Age 25 and under	21	84.00
	26–30 years old	4	16.00
Gender	Male	13	52.00
	Female	12	48.00
Education level	Undergraduate	14	56.00
	Graduate	8	32.00
	Ph.D. and above	3	12.00
Short music video preference (optional)	Traditional Chinese music	8	32.00
	Country music	5	20.00
	Modern pop music	13	52.00
	Western classical music	11	44.00
	Blues/Jazz	9	36.00
	Electronic/dance music	7	28.00

### Independent variables

Three independent factors were examined: rhythmic style, frame type (with two levels: narrative and non-narrative), and lyric relevance (with two levels: lyric relevant and lyric irrelevant) (two levels: 1—stable rhythm and 2—flow rhythm). The two that relate to the content of music videos are picture type and lyrics relevance. To make operation and analysis easier, we integrated the influencing factors for music video content into a single variable, music short video type, which has three levels:

1. Narrative short music video (with a complete story).
2. Live short music videos (Live performance, no plot).
3. Funny short music videos (Parody, no plot).
The rhythmic variable contains two levels:
1. Stable (mainly in 4, 2, and stable 3 time, mostly polka, march, pop music).
2. Flow type (mainly in 6-meter, 9-meter, or asymmetrical rhythm, moving freely and scattered).

### Dependent variable

Psychometric aspects of emotion:

(1) Wake up as determined by the SAM scale. The Likert scale has nine levels, with one representing the highest level of arousal, five the neutral level, and nine the lowest level.

Titer as determined on the SAM scale. The Likert scale at level nine, with one denoting positive titers (such as joy and happiness), five denoting neutral, and nine denoting negative titers (e.g., sadness, disappointment, and anger).

(2) As evaluated by the Geneva Musical Mood Scale, poetic. The Richter scale has five levels, with one denoting very inconsistent, three denoting neutral, and five denoting very consistent (17).

Dynamic as determined by the Geneva Musical Mood Scale. The scale included five levels, with one denoting a severe disagreement, three denoting neutral, and five denoting a strong agreement.

(3) Uncomfortable, according to the Geneva Musical Mood Scale. On a Likert scale of one to five, where one is highly negative, three is neutral, and five is very positive.

### Selection of short music video selection

The combination of various levels of independent variables was used to select the music videos that would be used in the study, asking four professionals with more than 4 years of music industry experience to rate the chosen music videos according to the strength of the independent variable factors they represent. Selection of short music video selection is shown in Table 2.

**Table 2:** Selection of short music video selection.

**Serial number**	**Name**	**Video content**	**Rhythm type**
1	<Little> by Rong Zuer	There's a whole storyline that goes with the lyrics	Stable rhythm
2	<Love like the Tide> by Zhang Xinzhe	There's a whole storyline that goes with the lyrics	Flow rhythm
3	<If One day> by Andy Lau	Live in concert	Stable rhythm
4	<Can't Talk> by Jay Chou	Live in concert	Flow rhythm
5	<The Most stringy National Wind> by Liu Meilin	Network funny short music video	Stable rhythm
6	<The Dance Of Joy> by the Rainbow cat and the Blue Rabbit	Network funny short music video	Flow rhythm

In addition, the subjects and music chosen for this study follow the following principles:

(1) The subjects made every attempt to become familiar with the songs to reduce the differences in familiarity levels. All subjects who were approved to participate in the experiment completed an online questionnaire survey in advance, and it was discovered that at least 60% of the music was recognizable to them.

(2) Music video integrity: Since strong emotional reactions depend on a range of circumstances and musical meanings, each brief music video, which usually lasts between three and 5 min, should be as inclusive as possible. Integrity is also demonstrated by the consistency of the elements: the vocal portion, accompaniment, and visual are all still present in the film together with all the other elements.

### Experiment design

The intra-group design mandated that each participant see all six music videos. Because the entire experimental process is straightforward and interesting, the consequences of weariness and load may be ignored. The in-group design's problem with the learning effect was resolved by the Latin square design, which has six sequential permutations and an equal chance for each dependent variable processing combination to appear at each location. In this study, 25 participants were randomly assigned to six sequential pairings.

Data analysis model:


$$\begin{array}{l} \text{Psychological emotion}=\mu +\text{short music videos type} \end{array}$$

(1) $$\begin{array}{l} +\text{RHYTHM}+\text{short music videos typ}{\text{e}}^{*}\text{RHYTHM}+\varepsilon \end{array}$$

μis the population mean; short music videos type refers to the fixed effect of music short video; *RHYTHM* refers to the rhythmic fixed effect; ε is a random effect.

Latin square design is shown in Table 3.

**Table 3:** Latin square design.

**Groups**	**Order**
	**1**	**2**	**3**	**4**	**5**	**6**
Group 1	A	B	C	D	E	F
Group 2	B	C	D	E	F	A
Group 3	C	D	E	F	A	B
Group 4	D	E	F	A	B	C
Group 5	E	F	A	B	C	D
Group 6	F	A	B	C	D	E

## Empirical results

### Reliability and validity tests

The four professionals with more than 4 years of professional music training evaluated the six songs and videos used in this experiment, and all of them passed. They strictly adhere to the corresponding independent variable level. This experiment made use of the SAM Mood Scale and the Geneva Musical Mood Scale (18). The Geneva Musical Mood Scale's internal consistency was examined using Cronbach's alpha. The used gemS-25 scale had measurement reliability scores of 0.874 for poetic, 0.929 for dynamic, and 0.709 for uneasy. In actual experiments, it is acceptable for the scale's internal consistency to rise above 0.6 if the measured value changes with the tasks. As a result, the three components of the Geneva musical mood scale will be included in the research's statistical model.

### Model fit test

The single sample Kolmogorov–Smirnov test was used to test the normality of the dependent variable data. The homogeneity test of variance is shown in Table 4.

**Table 4:** Homogeneity test of variance.

**Measure**	**Awaken**	**Poetic**	**Dynamic**	**Uneasy**	**Titer**
*F*	0.471	0.195	0.442	2.188	0.260
Sig.	0.822	0.985	0.308	0.083	0.956

The results showed that only poetry and vitality in the Geneva scale passed the normality test. Other psychometric data (Awaken, dynamic, and uneasy) still fail the normality test after the necessary conversion.

### Psychological data analysis

The impact of the type of music rhythm and brief music video on viewers' emotions was investigated using MANOVA (19). The independent variables in this model are the genre of music short video and the beat, whereas the dependent variables are poetry and energy (within the group). As a result, this approach does not account for individual differences. The non-parametric test method of Kruskal–Wallis and Mann–Whitney *U* was used in this work to conduct a one-way ANOVA for the arousal level, potency, and anxiety of emotions (20). In contrast to live performance short music videos, it is believed that narrative short music videos will elicit more emotions directly tied to emotional reaction. Narrative short music videos are thought to trigger more emotions associated with cognitive processing (like poetry). The expectation is that narrative short music videos will stir up more feelings than live short music videos (such as vitality). Hypothesis testing of narrative short music videos is shown in Table 5.

**Table 5:** Hypothesis testing of narrative short music videos.

**Dependent variable**		**Poetic**	**Dynamic**	**Uneasy**	**Awaken**	**Titer**
Narrative short music videos	Mean	3.068	2.735	2.269	4.581	4.265
	Std.	0.119	0.107	0.148	0.276	0.332
Live short music videos	Mean	2.645	3.609	1.548	4.731	4.422
	Std.	0.100	0.146	0.088	0.268	0.263
Funny short music videos	Mean	2.247	3.237	1.515	4.481	0.932
	Std.	0.102	0.134	0.112	0.279	0.273
*F*		32.088	22.126	25.689	0.789	18.011
Sig.		<0.05	<0.05	<0.05	>0.05	<0.05

The findings demonstrated that listening to humorous short music videos significantly increased listeners' positive valence emotions (happiness) compared to listening to narrative and live short music videos. Comparing the subjects' scores for the three brief music videos' potency and arousal levels revealed that while their overall potency was favorable, the scores for the videos' arousal levels were neutral. The potency and arousal levels, however, did not significantly differ between the live short music videos and the narrative short music videos. Funny short music videos are titillating, have a happy, joyful mood, and site-type music myopia bands, and give the viewers more of an associated dynamic mood. Narrative short music videos do induce more related to the poetic mood, and the plot contains emotional content that can cause psychological emotional resonance.

It is believed that while flowing rhythm can induce low arousal levels, which are more closely related to negative emotions, emotional, and poetic emotions, stable rhythm can induce high arousal levels, energetic, and positive emotions. The psychological and emotional impact of live short music videos on viewers is shown in Table 6.

**Table 6:** Psychological and emotional impact of live short music videos on viewers.

**Dependent variable**		**Poetic**	**Dynamic**	**Uneasy**	**Awaken**	**Titer**
Stable rhythm	Mean	2.600	3.602	1.537	4.115	2.715
	Std.	0.099	0.119	0.726	0.217	0.183
Flow rhythm	Mean	2.707	2.786	2.018	5.082	5.037
	Std.	0.101	0.110	0.118	0.223	0.245
*F*		1.896	66.926	10.932	10.829	48.018
Sig.		>0.05	<0.05	<0.05	<0.05	<0.05

The results showed that while awakening levels for the two rhythm types tended to be neutral, the awaken level of listeners who preferred the steady rhythm type was significantly higher than that of listeners who preferred the flow rhythm type. According to valence, steady rhythm significantly increases good feelings while a flowing rhythm significantly increases negative emotion. Fixed rhythm thus considerably increases viewers' emotional arousal level and promotes the growth of more favorable feelings. Stable rhythms induce cheerful, upbeat, and energizing feelings, whereas flow rhythms are more likely to elicit sad and dismal ones.

The results of the statistical test show that the interaction between funny short music video type and rhythm type has significant effects on the measured values of poetic emotion (*F* = 4.281, *P* < 0.05) and vitality emotion (*F* = 5.074, *P* < 0.05).

To further analyze the interaction effect between poetic and dynamic, this study conducted a simple effect analysis on three types of short music videos, respectively. The results are analyzed as shown in Table 7.

**Table 7:** Simple effect analysis of short music video.

**Dependent variable**		**Narrative short music videos**		**Live short music videos**		**Funny short music videos**	
		**Poetic**	**Dynamic**	**Poetic**	**Dynamic**	**Poetic**	**Dynamic**
Stable rhythm	Mean	3.257	3.471	2.309	3.609	3.726	3.726
	Std.	0.111	0.113	0.134	0.146	0.170	0.170
Flow rhythm	Mean	2.879	1.999	2.982	3.609	2.749	2.749
	Std.	0.138	0.144	0.128	0.146	0.167	0.167
*F*		15.045	92.764	18.065	–	25.996	25.996
Sig.		<0.05	<0.05	<0.05	–	<0.05	<0.05

Almost nothing in the amusing short music videos had any rhythmic impact on the participants' poetic feelings. While the participants were watching live short music videos, their sense of poetry revealed very substantial changes with distinct rhythm types: When subjects were viewing narrative short music videos, they felt more poetic when the beat was more steady; when they were watching live short music videos, they felt less poetic when the rhythm was more constant.

The individuals' sense of dynamic is essentially unaffected by the rhythm type when they watch the live short music videos. Short music videos significantly increased dynamic emotion when the individuals were watching narrative short music videos as opposed to humorous short music videos. This resulted from an improvement in rhythm stability. To put it another way, narratives might accentuate the potential for intense feelings to rise along with greater rhythmic steadiness.

### Correlation analysis of dependent variables

To further understand the psychological measures of music-video-induced emotion, this study conducted a correlation analysis of dependent variables. The results with strong correlation are listed in Table 8.

**Table 8:** Correlation analysis of dependent variables.

**Psychological**	**Normalized**	**Sig**.
**emotions**	**Pearson coefficient**	
Poetic	−0.75	0.047
Dynamic	−0.205	0.026
Uneasy	0.233	0.018
Awaken	0.184	0.038
Titer	−0.166	0.058

The outcomes demonstrated that, in contrast to music-induced emotions, unease played a dominant role in those emotions. Emotion valence and short music video type showed a significant positive correlation. A close relationship exists between the brief video of a sense of vitality music and a particular musical mood.

### Hypothesis testing results

The verification of all hypotheses is summarized in this study, and the hypothesis testing results are shown in Table 9.

**Table 9:** Hypothesis testing results.

**Hypothesis**	**Content**	**Authentication**
1	The emotional valence induced by narrative short music videos is more polarized than performance short music videos	Authorized
2	The emotional arousal level induced by narrative short music videos is more polarized than performance short music videos	Unauthorized
3	The type of short music videos has an effect on the specific types of emotions induced by short music videos	Authorized
4	Rhythm type has an effect on the emotion induced by live short music videos. Steady rhythm has more positive valence and higher arousal level than flow rhythm	Authorized
5	Rhythmic type has an effect on specific emotions induced by live short music videos. Stable rhythm and flow rhythm induce different types of specific musical emotions	Part authorized
6	Funny short music videos and rhythm have an interaction on psychological emotions	Authorized
7	Funny short music videos interact with rhythmic music for specific emotions	Authorized

### Discussion on the result

The results of this study are summarized as follows:

(1) Influence of short music video type

Different types of music short videos can elicit emotional reactions of varying intensity. In terms of emotional valence, narrative short music video ratings are typically more divisive than live short music video ratings. Storylines do not require as much fine processing, so people are more likely to process information along the edges, which intensifies their emotional reactions. Short performance music videos, on the contrary, tend to be more abstract and may call for more delicate cognitive processing. The short live music video induces a lower level of poetic emotion than the short narrative music video, which is more sensitive to poetic emotion.

(2) Funny music short video-induced emotions

Funny short music films cause various types and levels of emotions than standard short music videos do. Compared to the narrative and live short music videos, the highly entertaining content made the subjects laugh and they tended to score favorably on the valence scale. According to psychological, physiological, and experimental observation data, when people like the humorous music short video used in this study, they experience strong feelings of happiness and excitement.

(3) Music short video type is influenced by rhythm type

The results of the hypothesis testing indicate that various types of music-specific short videos are responsive to various emotions. Poetry-related rhythm types respond to narrative and live music short videos for poetic emotion, but not to funny music short videos. While flowing rhythm produced a more poetic mood than steady rhythm in a short live music video, steady rhythm produced a more poetic mood in a short narrative music video. While live music videos are almost never affected by rhythm, the first two categories are higher and will be affected by rhythm for dynamic emotions in narrative and funny short music videos. The atmosphere, the performance, and the movements of the singers in short live music videos based on live performance and audience interaction energized the subjects more than the music itself did (in this study).

## Conclusion

According to the findings of this study, the type of short music video significantly affects the valence of emotion, as does the interaction between the type of short music video and musical characteristics. Compared to narrative and live music short videos, funny music short videos significantly increased the positive emotion in viewers. In comparison with that, the neutral emotion valence induced by the narrative and live music short video was significantly higher. Performance music short videos produced more polarized emotional valence than narrative music short videos. The emotional valence, arousal, vitality, and restlessness in a brief music video were significantly influenced by the rhythm type. This study encountered some issues while investigating the impact of different types of music videos on musically induced emotions. The influence of personality, age, and gender on musical emotion perception has been discussed in studies on individual differences in musical emotion perception, but previous findings have not produced significant effects in this study due to time and energy constraints. Future research can begin from two angles: first, experiment on people of different ages and backgrounds; and second, conduct group experiments to see whether the emotions elicited by pop music and classical music with the same musical characteristics were influenced by personal differences and musical background.

## Data availability statement

The original contributions presented in the study are included in the article/supplementary material, further inquiries can be directed to the corresponding author.

## Ethics statement

Written informed consent was obtained from the individual(s) for the publication of any potentially identifiable images or data included in this article.

## Author contributions

LM completed relevant research work on the manuscript and agreed to publish it in this journal.

## Conflict of interest

The author declares that the research was conducted in the absence of any commercial or financial relationships that could be construed as a potential conflict of interest.

## Publisher's note

All claims expressed in this article are solely those of the authors and do not necessarily represent those of their affiliated organizations, or those of the publisher, the editors and the reviewers. Any product that may be evaluated in this article, or claim that may be made by its manufacturer, is not guaranteed or endorsed by the publisher.
